# Reference genes identification for qRT-PCR normalization of gene expression analysis in *Cucumis sativus* under *Meloidogyne incognita* infection and *Pseudomonas* treatment

**DOI:** 10.3389/fpls.2022.1061921

**Published:** 2022-12-15

**Authors:** Tingting Ji, Si Ma, Meiting Liang, Xueyun Wang, Lihong Gao, Yongqiang Tian

**Affiliations:** Beijing Key Laboratory of Growth and Development Regulation for Protected Vegetable Crops, College of Horticulture, China Agriculture University, Beijing, China

**Keywords:** *Cucumis sativus*, qRT-PCR, reference gene, *Meloidogyne incognita*, *Pseudomonas*

## Abstract

qRT-PCR is a common and key technical means to study gene expression in biological research. However, reliability and accuracy of quantification by qRT-PCR is entirely dependent on the identification of appropriate reference genes. Cucumber as an economical vegetable is widely cultivated worldwide and is subject to serious nematode infection, especially from *M. incognita*. Plant could employ beneficial soil bacteria in the rhizosphere to enhance plant adaptability to various stresses. In this study, the optimal reference genes in cucumber under *M. incognita* stress and *Pseudomonas* treatment were calculated and confirmed. A total of thirteen candidate reference genes were identified across three different treatments. Of these, geNorm, NormFinder and BestKeeper programs combined RefFinder software identified *EF1* and *UBI* are the most suitable reference gene in the root knot and whole root of cucumber infected *M. incognita*, respectively, and *CACS* is the most suitable reference gene in the whole root of cucumber treated by *Pseudomonas*. The work first validated the most suitable reference genes for the normalization gene expression in cucumber by nematode infected or *Pseudomonas* inoculated, and these results would facilitate the further research on *M. incognita* or *Pseudomonas* soil rhizosphere microbe interaction with cucumber.

## Introduction

Cucumber (*Cucumis sativus* L.) is one of the most important vegetable crops in protected cultivation, which is widely planted in the world. Cucumber has been developed as a new model species in plant biology due to its many desirable traits, including (i) small number of genes, (ii) rich diversity of sex expression, (iii) suitability for vascular biology studies short life cycle (three months from seed to seed), (iv) mixed phloem loading mechanism (Ma et al., 2019), and (v) *Agrobacterium tumefaciens*-mediated transformation (Li et al., 2017). The physiological, biochemical and breeding of cucumber have been studied for several decades, and it became more realistic to further study the molecular biology of cucumber as available of genomic sequence of cucumber (http://cucurbitgenomics.org/organism/2) (Huang et al., 2009) and accumulating resources in genetics and genomics.

In recent years, it has been found that root-knot nematode has caused serious damage to protected cultivation vegetable and leads to the continuous production reduction of cucumber in most areas due to growth obstacles (Jaiteh et al., 2012). So far, four kinds of root knot nematodes, including *Meloidogyne javanica* (*M. javanica*), *Meloidogyne arenaria* (*M. arenaria*), *Meloidogyne incognita* (*M. incognita*), and *Meloidogyne hapla* (*M. hapla*) are considered as serious threats to crop productions, among them, *M. incognita* has the widest distribution of host (Moens and Perry, 2009; Shahid et al., 2022). As its obligate biotrophic nature, *M. incognita* mainly relies on the host plant for nutrition and maintains the relationship with the host in a few weeks (Jones et al., 2011; Yook et al., 2011). Besides, *M. incognita* affects the root activity and the root absorption and transportation of water and inorganic salt ions, and hormones content like JA, SA and ABA, resulting in short aboveground plants, abnormal yellowing of leaf color, defoliation, growth weakness, wilting, and reduced yield (Mbaluto et al., 2020). There has been a lot of research on plant-nematode interaction, including how nematodes find their host, how the host perceive nematode precontact, and which host defense responses are elicited upon perception. Thus, many regulated genes have been reported in response to nematode infection, such as transporter genes, hormone related genes and cell wall embolism gens. *AtACA8* (P-type ATPase gene), *AtAUX1* (auxin influx transporter gene) and *AtSUC1* (sucrose transporter gene) were induced significantly in Arabidopsis after infected by *M. incognita* though microarray analysis (Hammes et al., 2005). In rice, *OsBAK1* (brassinolide co-receptor gene) was induced by *M. incognita* infection based on the RNA-Seq analysis (Zhou et al., 2020). Although these genes expression have also been identified by qRT-PCR, there has been no selection research of housekeeping genes (HKGs) in plant infected by *M. incognita* yet.

Plant growth-promoting rhizobacteria (PGPR) is a kind of important beneficial bacteria that promote plant growth and development, absorb nutrients, improve plant stress and inhibit pests (Vejan et al., 2016). Plants inoculation with PGPRs stains, such as *B. subtilis GB03* and *Pseudomonas* spp., could enhance the tolerance for osmotic stress in plants by up-regulate the glycine betaine biosynthesis (Sandhya et al., 2010; Zhang et al., 2010). PGPR indirectly boosts plant growth rate, has also been widely reported in the study of microbial plant interaction. *Pseudomonas* is an important PGPR that promotes seed germination, root growth, accumulation of mineral nutrients, water use and prevention of plant diseases. Under salinity conditions, *Pseudomonas* was enriched in the rhizosphere and endosphere to enhance plant adaptability to salt stress through inducing the production of stress alleviating metabolites, like indole acetic acid, exopolysaccharides, and gibberellins. In this context, many functions of *Pseudomonas* on plants have been examined in physiology view (Kumar et al., 2019; Li et al., 2021). However, there is little research involving the molecular mechanism about the interaction between *Pseudomonas* and plants. Thus, the identification of appropriate reference genes will be valuable for advancing the plant-microorganism interaction.

Gene expression analysis is the basis for elucidating the molecular mechanisms of various biological processes, and quantitative real-time polymerase chain reaction (qRT-PCR) has become a common method to study gene expression due to its advantages of good repeatability, high sensitivity, high specific and high throughput (Nolan et al., 2006). However, this approach requires one or more HKGs that are stably expressed as criteria for normalizing gene expression. The ideal HKGs should be systematically evaluated in different tissues under different experimental conditions, and its expression should be stable and then used as a control for qRT-PCR analysis (Nolan et al., 2006). HKGs are a class of stably expressed genes, which can maintain the basic functions of cell division, growth and development, cell apoptosis and the whole physiological process of plant metabolism. Many HKGs, including *Actin* (*ACT*), *a-Tubulin* (*TUA*), *F-box protein* (*F-BOX*), *YSL8* (*mitosis protein*), *Ubiquitin-conjugating enzyme E2* (*UBC*), 6*0S ribosomal protein L36a/L44* (*PRL36Aa*), *Protein phosphatase* (*PP2A*) and *Clathrin adaptor complexes medium submit family protein* (*CACS*) had been used in cucumber (Migocka and Papierniak, 2011; Warzybok and Migocka, 2013; Joseph et al., 2018; Liang et al., 2018; Miao et al., 2019). After metal treatment, *CACS* expression was the most stable reference gene, and *EF1* expression was the most reliable reference gene under salt, osmotic and oxidative stresses in cucumber (Migocka and Papierniak, 2011). Under different nitrogen conditions, *TIP41*, *F-box* and *EF1* were the most suitable genes to normalize *CsNRTs* expression (Warzybok and Migocka, 2013). For Cucumber (*Cucumis sativus* L.), pumpkin (*Cucurbita moschata* Duch.) and cucumber-pumpkin grafting experiment, *CACS* and *40SRPS8* were the most stable reference genes in all samples (Miao et al., 2019). A mass of experiments demonstrated that HKGs cannot be utilized universally across different experimental conditions, plant species, developmental stages or even different organs within a single species. Therefore, many researchers have tried to find reliable reference genes expressed under specific experimental conditions and multi-HKGs should be used in one experiment.

Here, in order to promote the current quantification of gene expression in cucumber roots infected *M. incognita* and treated by *Pseudomonas*, thirteen candidate HKGs across seven timepoints in cucumber roots, which were infected by *M. incognita* and inoculated by three *Pseudomonas*, were assessed to identify suitable cohort of stably expressed HKGs for accurate and reliable normalization of cucumber qRT-PCR expression data, respectively. According to four procedures and some statistical methods, including geNorm (Vandesompele et al., 2002), NormFinder (Andersen et al., 2004), BestKeeper (Pfaffl et al., 2004), and RefFinder (Duan et al., 2017), the most suitable candidate HKGs were finally screened. The selected suitable reference genes will be helpful for understanding the cucumber-nematode interaction and investigating how *Pseudomonas* to improve plant growth on molecular level.

## Materials and methods

### Plant material and treatments

Cucumber (*Xintaimici*) was used in this study. The cucumber seeds were germinated in 25°C of darkness and sown in black pots with soil and sand (v:v, 1:1). Seedlings were grown a 75%-90% relative humidity and 16 h photoperiod (200 mmol m^-2^ s^-1^) at 28°C/day and 18°C/night for 7 days with standard Hoagland nutrient solution. Two treatments were applied in this study, which were *M. incognita* infection and *Pseudomonas* inoculation, respectively. Plant samples were divided into three categories, root knots produced by *M. incognita* (Treatment 1), whole root infected by *M. incognita* (Treatment 2) and inoculated by *Pseudomonas* (Treatment 3).

Nematode egg masses were collected from infested swamp cabbage roots and kept in sterile water for hatching at 28°C. Three-week-old cucumber seedlings were inoculated with freshly hatched second-stage juvenile nematode and 500 nematodes per plant. Cucumber roots infected by *M. incognita* (root knots and whole roots) were harvested at 0 d, 7 d, 14 d, 21 d, 28 d, and 35 d. For the *Pseudomonas* treatment, RH58, RH61, and RH62 strains were used, the purified beneficial rhizobacteria strains were shaken to OD_600 =_ 1.0. The roots were collected at inoculated 72 h by *Pseudomonas*.

### Total RNA isolation and cDNA synthesis

Cucumber root tissues were disrupted under liquid nitrogen frozen conditions. Approximately 100 mg of the ground root tissues were placed into a 1.5 mL centrifuge tube and total RNA extracted using RNeasy plant kit (Huayueyang, Beijing, China). RNA concentration and purity were examined by nucleic acid spectrophotometer (NanoDrop™ 1000, ThermoFisher Scientific, USA) and OD_260/280_ showed values between 1.8 and 2.0. The quality of RNA was detected by agarose gel electrophoresis. 1 ug of total RNA was used for cDNA synthesized using Fastking cDNA Dispelling RT Super Mix Kit (TIANGEN, Being, China) according to the manufacturer’s instructions. cDNA was diluted 1:5 in RNase-free water and stored at -20°C until use in qRT-PCR experiment.

### Candidate reference genes selection and qRT-PCR assay

**Figure 1:**
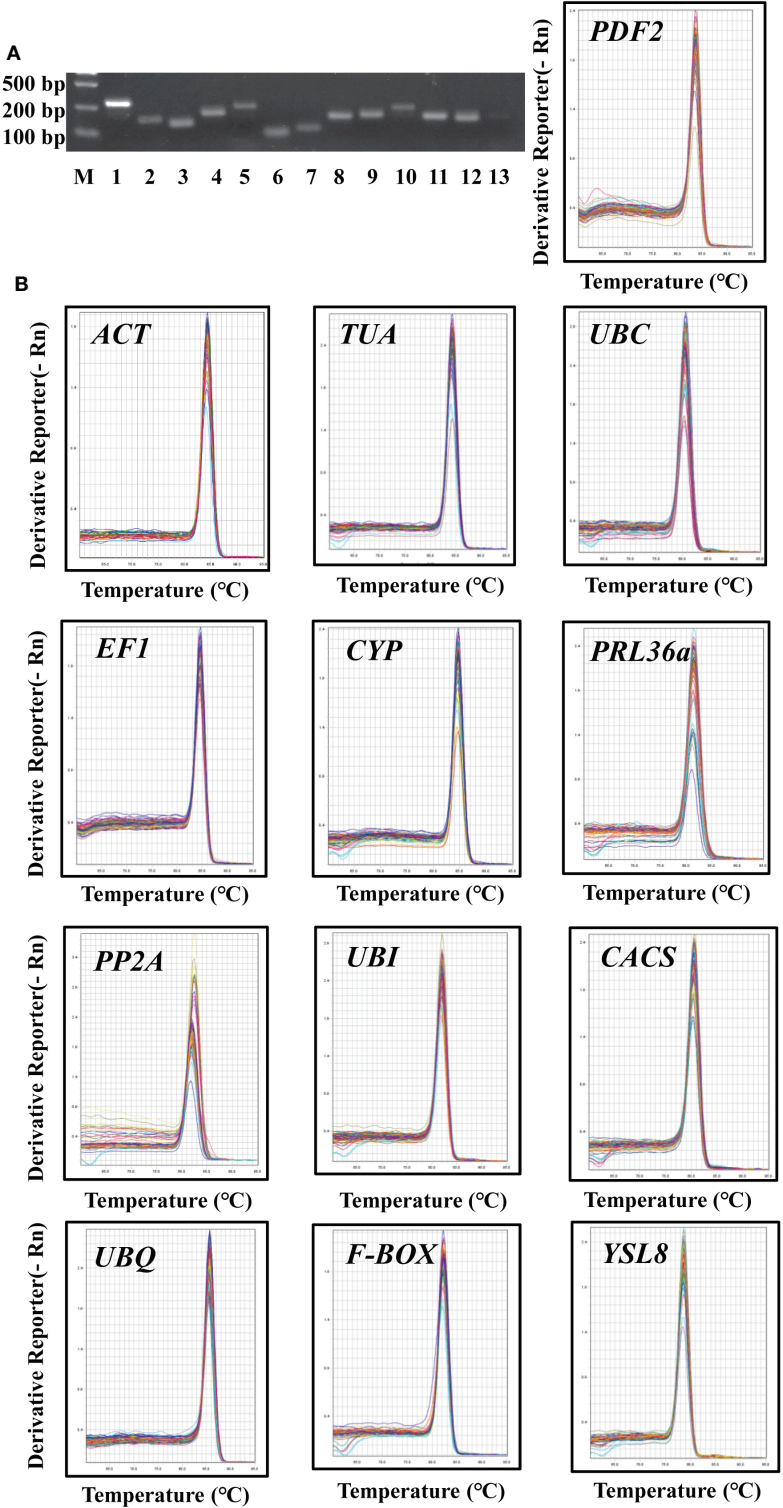
Primer specificity of candidate reference genes. **(A)**, Agarose gel analysis of RT-PCR generated amplicons for each of the thirteen assessed candidate reference genes. M, marker; 1-13, *ACT*, *TUA*, *UBC*, *EF1*, *CYP*, *PRL36Aa*, *PP2A*, *UBI*, *CACS*, *UBQ*, *F-BOX*, *YSL8*, PDF2. **(B)**, Melt curve analysis of the thirteen assessed candidate reference genes across different samples in cucumber roots showed a single peak for each primer pair at a specific annealing temperature.

A total of thirteen potential reference genes were selected in this study. According to previous studies, the expression of these candidate genes is relatively stable in plant growth and development processes, or under biotic or abiotic stresses. The sequences of candidate reference genes were acquired from the GenBank and Cucumber genome database. The primer pairs (**Table 1**) were completely consistent with previous study (Migocka and Papierniak, 2011; Miao et al., 2019). The primers specificity of thirteen candidate reference genes were detected by PCR and 1% agarose gel electrophoresis (**Figure 1**). qRT-PCR experiment was performed on an ABI 7500 Real Time PCR system (Applied Biosystems, USA) using SYBR^®^ Green I (ChamQ SYBR qPCR Master Mix) (Vazyme, Beijing, China). Each 10 μl reaction mixture contained 2 μl of cDNA template, 5 μl of SYBR^®^ Green I, 0.2 μl of each primer, and 2.6 μl of ddH_2_O. The qRT-PCR reaction system was as follow: 94°C for 30 s, 40 cycles of 94°C for 5 s and 60°C for 34 s.

**Table 1:** Cucumber reference genes information and their primer sequences.

Gene	Accession number	Annotation	Gene ID in cucumber	Forward primer	Reverser primer	References
*ACT*	AB010922	Actin	Csa6G484600	CCGTTCTGTCCCTCTACGCTAGTG	GGAACTGCTCTTTGCACTCTCGAG	Niu et al., 2018; Wen et al., 2019; Zhang et al., 2021; Qin et al., 2021
*TUA*	AJ715498	Alpha-tubulin	Csa4G000580	CATTCTCTCTTGGAACACACTGA	TCAAACTGGCAGTTAAAGATGAAA	Lü et al., 2017; Sun et al., 2019
*UBC*		Ubiquitin conjugating enzyme	Csa3G358610	GTCACCATTCATTTTCCTCCG	GGGCTCCACTGCTCTTTCA	Song et al., 2019
*EF1*	EF446145	Elongation factor 1-alpha	Csa2G139820	ACTTTATCAAGAACATGATTAC	TTCCTTCACAATTTCATCG	Wang et al., 2015; Zhang et al., 2019
*CYP*	AY942800	Cyclophilin	Csa7G009740	TTTCATGTGCCAGGGAGG	AGCCAATCGGTCTTAGCG	Le et al., 2012; Olaetxea et al., 2015
*PRL36Aa*	HM594174	60S ribosomal protein L36a/L44	Csa3G653380	AAGATAGTCTTGCTGCACAGGG	AACACGGGCTTGGTTTGA	Miao et al., 2019
*PP2A*	HM594171	protein phosphatase 2A regulatory subunit A	Csa5G608520	GAAGCTGTAGGACCTGAACCA	AGCCGCTGCAATACGAAC	Petriccione et al., 2015
*UBI*	AF104391	Ubiquitin-like protein	Csa2G036600	CCTTATTGACCAACCAGTAGT	GGACAATGTTGATTTCCTCG	Wen et al., 2019; Zhao et al., 2019; Shen et al., 2019
*CACS*	GW881874	Clathrin adaptor comple subunit	Csa3G902930	TGGGAAGATTCTTATGAAGTGC	CTCGTCAAATTTACACATTGGT	Migocka et al., 2011; Rajsz et al., 2016
*UBQ*	AY372537	Polyubiquitin	Csa4G089780	CACCAAGCCCAAGAAGATC	TAAACCTAATCACCACCAGC	Wei et al., 2016; Kopczewski et al., 2022
*F-BOX*	GW881870	F-box protein	Csa5G642160	GGTTCATCTGGTGGTCTT	CTTTAAACGAACGGTCAGTCC	Rodrigues et al., 2010; Le et al., 2012
*YSL8*	GW881872	mitosis protein	Csa5G175720	CCTTGTGGATATCACAGAAGTT	CTTGTTTATCCTTGAGTGCC	Song et al., 2019
*PDF2*	GW881868	Protein phosphatase 2	Csa5G608520	GTAGGACCTGAACCAACTA	CTTCACGCAGGGAAGA	Chen et al., 2017

### Gene expression stability analysis

A boxplot of cycle threshold (CT) values to evaluate the expression levels of thirteen candidate reference genes. To evaluate the expression stability of thirteen candidate reference genes under different experiment treatments, four statistical algorithms, including geNorm (Vandesompele et al., 2002), NormFinder (Andersen et al., 2004), BestKeeper (Pfaffl et al., 2004), and RefFinder, were used. In the geNorm program, M value represents the expression stability of calculated genes, and V value represents the pair average variation of measured genes. The lower the M value of the reference gene, the higher expression stability, and the M value ≥1.5 indicates that the expression of candidate genes is unstable (Vandesompele et al., 2002). The NormFinder program firstly obtains the expression stability value of each gene, then selects the most appropriate reference gene according to the stability value of candidate genes (Andersen et al., 2004). BestKeeper can be used to compare the expression levels of 10 candidate reference genes in 100 samples at most. BestKeeper can calculate the standard deviation (SD) and coefficient of variation (CV) of each gene and determine the expression stability of candidate genes by comparing SD and CV (Pfaffl et al., 2004). Finally, a comprehensive ranking of candidate genes was produced by RefFinder (Duan et al., 2017). RefFinder is a web-based tool that integrates geNorm, Normalfinder, BestKeeper and ΔCT method program, which provides the overall ranking of candidate genes through geometric average of attributed weights of each algorithm.

## Result

### Primer specificity of the candidate reference genes

In order to detect the specificity of primers, all primers were tested using RT-PCR approach and the construction of melt curves. For each prime pair, a single amplicon was observed by agarose gel analysis (**Figure 1A**). The melting curve of DNA refers to the curve of the degradation degree of DNA double helix structure with the increase of temperature. The melting curve was generated by monitoring the fluorescence signal. Different DNA sequence has different melt temperature (Tm) value, so it represents the specificity of amplification products. In this study, the amplification Tm value of each candidate gene was analyzed by qRT-PCR, thirteen candidate genes had specific characteristic peak at melting curve, respectively (**Figure 1B**). These results indicated that each primer pair of thirteen candidate genes was specific to targeted region.

### Expression analysis of candidate reference genes

**Figure 2:**
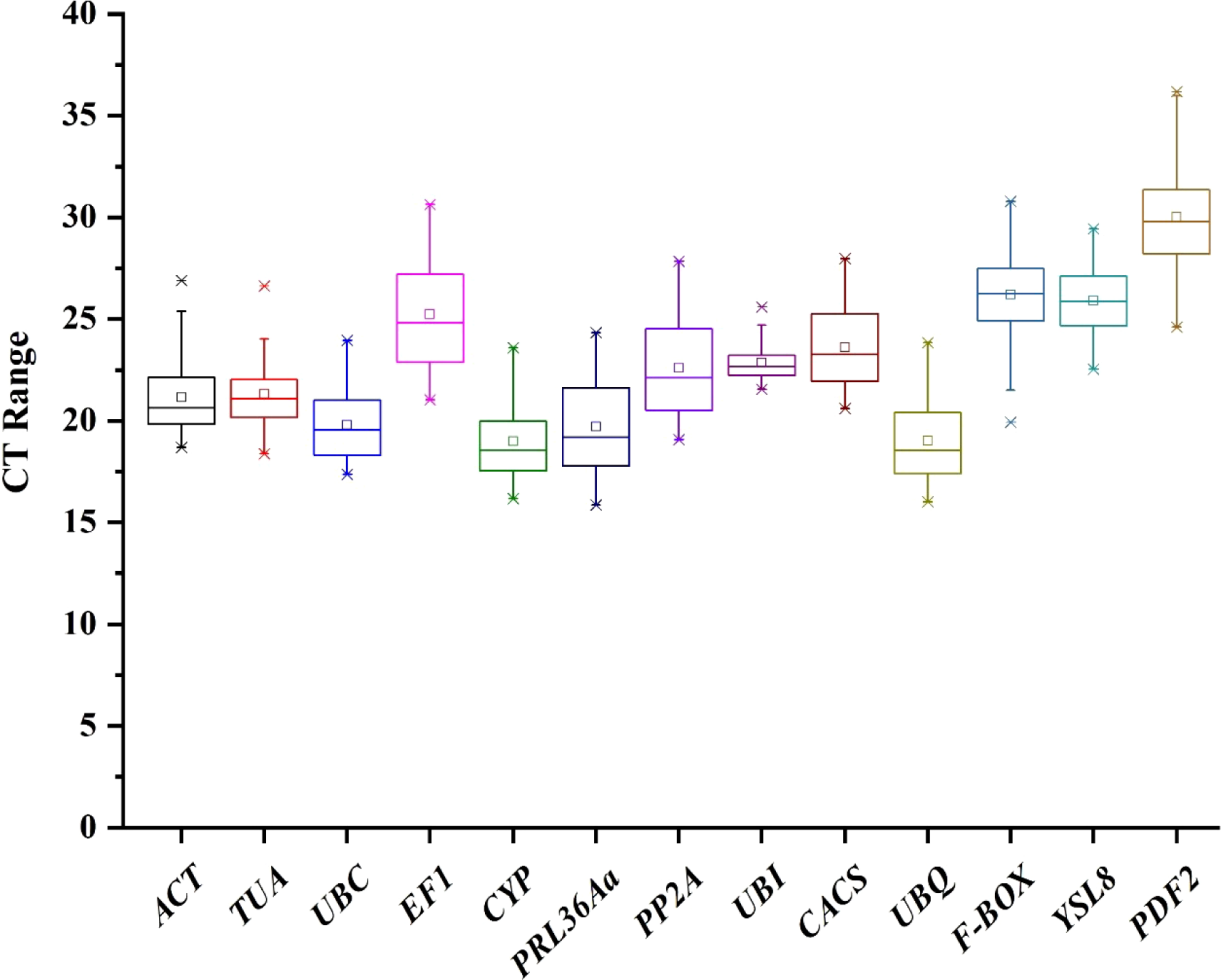
The thirteen candidate reference genes quantification cycle distributions.

In order to evaluate the expression stability of thirteen candidate genes, expression change of candidate genes under three experimental conditions were detected: Treatment 1, cucumber root knots were sampled after infected by *M. incognita*; Treatment 2, cucumber whole roots were sampled after infected by *M. incognita*; Treatment 3, cucumber whole roots were sampled after inoculated by *Pseudomonas*. The quantification cycle (Ct) values of thirteen candidate genes were used for data analysis in **Figure 2**. Ct value indicates the transcriptional quantity and with a lower Ct value indicating a more abundant target transcript. The Ct values of thirteen candidate genes in three treatments were calculated, respectively (**Supplement Table 1**). In treatment 1, *CYP* with the lowest mean Ct value (17.54), 28.11 for *PDF2* with the highest mean Ct value, and other eleven gens were primary distributed between 17 and 25. In treatment 2, similar trend was found that *CYP* and *PDF2* with the lowest (20.38) and highest (30.98) average Ct value, respectively. However, in treatment 3, the smallest mean Ct value was 17.76 for *UBQ* and the highest mean Ct value 32.37 for *PDF2*. The box plot in **Figure 2** provides an expression level overview of thirteen candidate genes combining three treatments, the results showed that the mean Ct value varied from 18.99 for *CYP*, to 30.01 for *PDF2*.

### The expression stability of candidate reference genes

**Figure 3:**
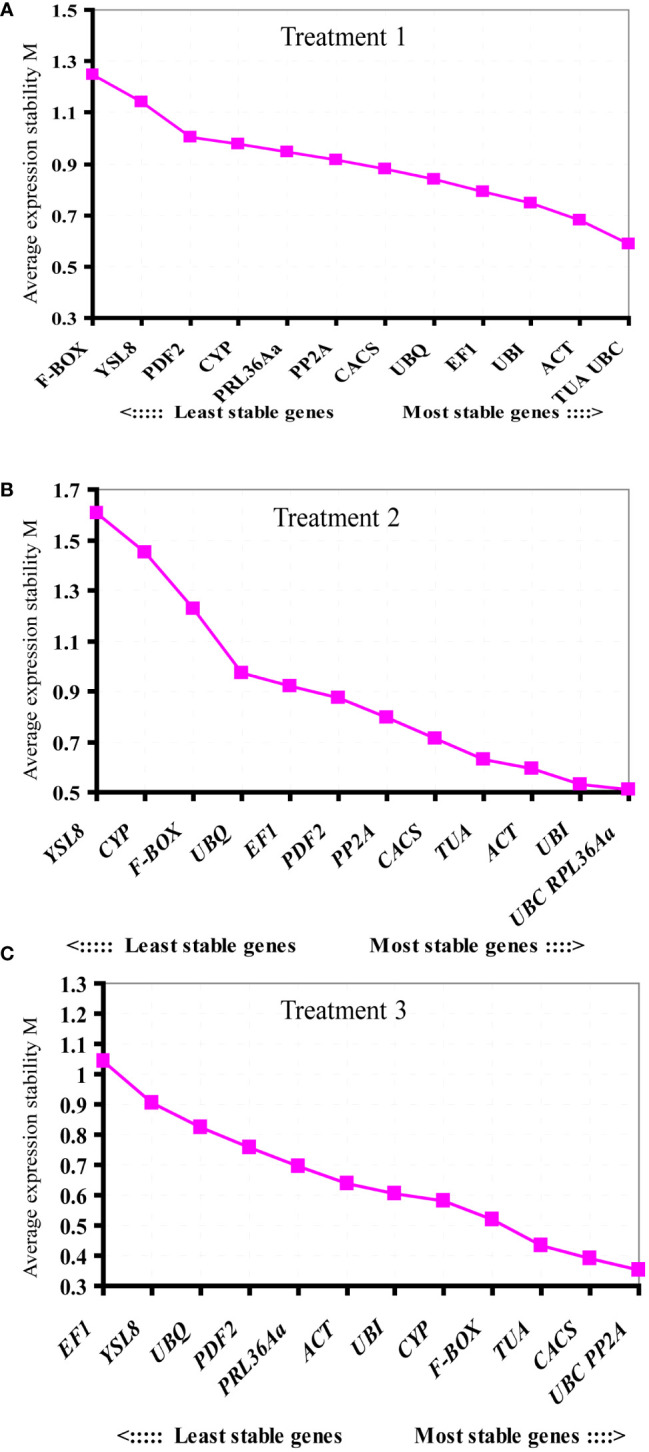
Ranking of the candidate reference genes according to geNorm analysis. The M value showing gene expression stability was calculated in three different treatments. **(A)** root knot treated by *M. incognita* (Treatment 1), **(B)** whole root treated by *M. incognita* (Treatment 2), and **(C)** whole root treated by *Pseudomonas* (Treatment 3).

**Figure 4:**
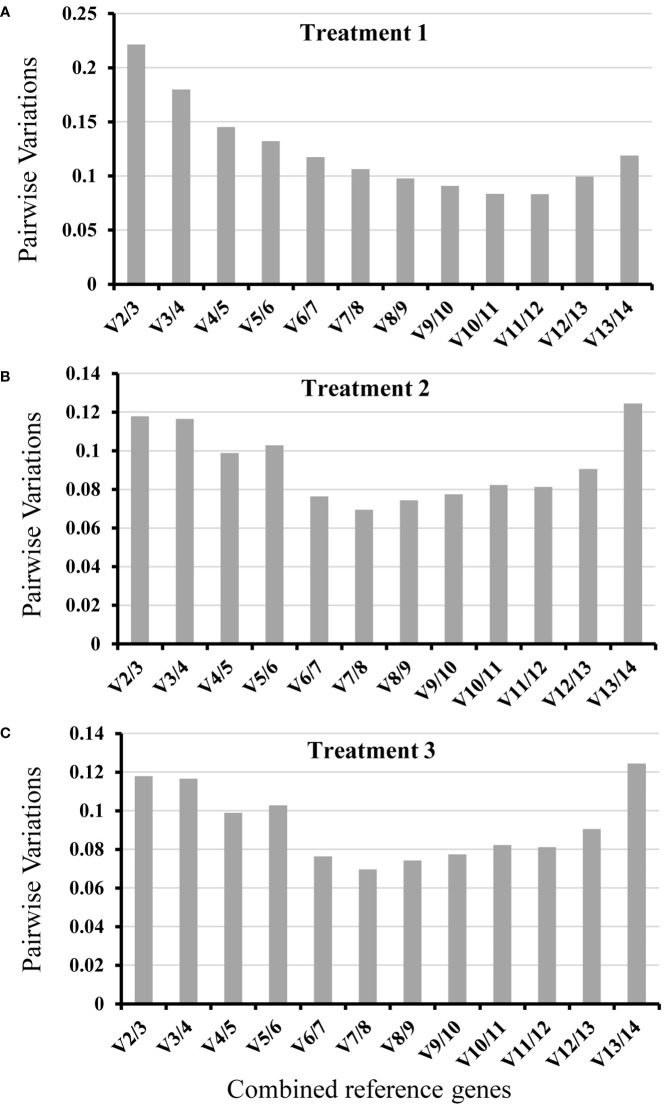
Pairwise variation (V) of candidate reference genes calculated by geNorm. The Vn/Vn+1 value calculated reflecting gene expression stability of thirteen candidate reference genes in three different treatments. **(A)**, Treatment 1: root knot samples caused by *M. incognita*; **(B)**, Treatment 2: whole root infected by *M. incognita*; **(C)**, Treatment 3: whole root treated by *Pseudomonas*.

To identify the most suitable reference genes with stable expression across all assessed samples in experiment, three frequently used statistical algorithms, containing geNorm, NormFinder and BestKeeper, were applied. geNorm evaluates reference gene average suitability value (M) based on the relative quantitative data (2^-ΔCt^, ΔCt = each corresponding Ct value - the minimum Ct value) of each candidate gene (Vandesompele et al., 2002). The lower value of M reflected more stable gene expression. In treatment 1, based on the geNorm program, the stability ranking of 13 tested candidate genes was: *TUA/UBC* > *ACT* > *UBI* > *EF1* > *UBQ* > *CACS* > *PP2A* > *PRL36Aa* > *CYP* > *PDF2* > *YSL8* > *F-BOX* (**Figure 3A**). Among the thirteen candidate genes, *TUA* and *UBC* genes with low M values were the most stable candidate genes, while of *F-BOX* gene with high M values was the most unstable candidate gene compared with the other twelve genes. For whole roots infected by nematode (Treatment 2), the order of gene expression stability was: *UBC*/*RPL36Aa* > *UBI* > *ACT* > *TUA* > *CACS* > *PP2A* > *PDF2* > *EF1* > *UBQ* > *F-BOX* > *CYP* > *YSL8* (**Figure 3B**). *PL36Aa* and *UBC* genes were the most stable genes, but the expression of *YSL8* was unstable as the M value ≥ 1.5 (Vandesompele et al., 2002), thus *YSL8* was not suitable as a reference gene in this treatment. For *Pseudomonas* treatment (Treatment 3), the order of gene expression stability was: *UBC*/*PP2A* > *CACS* >*TUA* > *F-BOX* > *CYP* > *UBI* > *ACT* > *RPL36Aa > PDF2* > *UBQ* > *YSL8* > *EF1* (**Figure 3C**). Based on the M values, *UBC* and *PP2A* were the most stable genes, while *EF1* was the worst one compared with the other twelve candidate genes. The pairwise variation value (Vn/Vn+1) could be calculated by geNorm, which determined the optimal number of reference genes. According to Vandesompele et al. (2002), Vn/Vn+1<0.15 indicates the optimal number of reference genes is achieved. In treatment 1, except for V2/3 and V3/4, the other value of Vn/Vn+1<0.15, V2/3 was already below the threshold in treatment 2 and treatment 3 (**Figure 4**).

Another program for analyzing the stability of reference genes, NormFinder uses a mathematical model to describe the expression values measured by RT-PCR, analyzes the sample subgroups separately, estimates the expression changes within and between groups, and then synthesizes into a stable value, that is the stable value of candidate gene. The judgment standard is that the most appropriate candidate gene showed minimum value calculated by NormFinder. In treatment 1, based on the NormFinder program analysis, the stability sequence of the thirteen tested candidate genes was: *EF1* > *UBQ* > *UBI* > *ACT* > *CACS* > *UBC* > *CYP* > *TUA* > *PRL36Aa* > *PP2A* > *PDF2* > *YSL8* > *F-BOX* (**Table 2**). Among the thirteen genes, *EF1* was the most stable gene with the lowest value 0.357, and *F-BOX* was the most unreliable one. In treatment 2, the expression stability of candidate gene was: *UBI* > *ACT* > *CACS* > *UBC* > *PRL36Aa* > *TUA* > *PP2A* > *UBQ* > *PDF2* > *EF1* > *F-BOX* > *CYP* > *YSL8* (**Table 2**). The results showed that *UBI* was the most reliable gene with the lowest value of 0.278, and the maximum value of *YSL8* was 1.589, which was the most unreliable gene compared with the other candidate genes. In treatment 3, the sequence of candidate gene expression stability was: *CACS* > *UBC* > *PP2A* > *TUA* > *CYP* > *F-BOX* > *UBI* > *ACT* > *PRL36Aa* > *PDF2* > *UBQ* > *YSL8* > *EF1* (**Table 2**). Among the thirteen genes, *CACS* with the minimum value 0.102 suggested it was the most reliable gene, while the value of *EF1* was the highest (1.201).

**Table 2:** Ranking and expression stability values of candidate genes by NormFinder.

Ranking order	Treatment 1		Treatment 2		Treatment 3	
	Gene name	Stability value	Gene name	Stability value	Gene name	Stability value
1	*EF1*	0.359	*UBI*	0.278	*CACS*	0.102
2	*UBQ*	0.374	*ACT*	0.377	*UBC*	0.221
3	*UBI*	0.419	*CACS*	0.406	*PP2A*	0.299
4	*ACT*	0.463	*UBC*	0.470	*TUA*	0.327
5	*CACS*	0.482	*PRL36Aa*	0.506	*CYP*	0.342
6	*UBC*	0.492	*TUA*	0.573	*F-BOX*	0.371
7	*CYP*	0.503	*PP2A*	0.594	*UBI*	0.408
8	*TUA*	0.547	*UBQ*	0.685	*ACT*	0.456
9	*PRL36Aa*	0.574	*PDF2*	0.820	*PRL36Aa*	0.521
10	*PP2A*	0.606	*EF1*	0.823	*PDF2*	0.541
11	*PDF2*	0.618	*F-BOX*	1.393	*UBQ*	0.689
12	*YSL8*	1.113	*CYP*	1.584	*YSL8*	0.835
13	*F-BOX*	1.200	*YSL8*	1.589	*EF1*	1.201

BestKeeper program can only compare the expression levels of up to 10 candidate genes in 100 samples, generally, the analysis of candidate genes by BestKeeper program is based on geNorm program and NormFinder. The criterion of BestKeeper program for determining the stability of candidate genes was that the smaller SD (Standard Deviation) and CV (Coefficient of variation), the better stability. When SD > 1, the expression of candidate gene was unstable. Based on geNorm Program and NormFinder analysis, the top-ranked ten candidate genes were selected for further analyzing by BestKeeper. In treatment 1, top ten candidate genes, including *UBI*, *PRL36Aa*, *ACT*, *UBQ*, *CACS*, *EF1*, *UBC*, *PP2A*, *CYP* and *TUA*, were selected for further analysis. In treatment 2, ten genes, including *ACT*, *TUA*, *UBC*, *EF1, RPL36Aa*, *UBI*, *PP2A*, *CACS*, *PDF2*, *UBQ*, were selected for BestKeeper analysis. In treatment 3, ten candidate genes including *ACT*, *TUA*, *UBC*, *CYP*, *PRL36Aa*, *PP2A*, *UBI*, *CACS*, *F-BOX*, *PDF2* for further study (**Table 2**, **Table 3**).

**Table 3:** Candidate genes and their expression stability values calculated by BestKeeper.

Treatment 1			Treatment 2			Treatment 3		
gene	SD	CV [% CP]	gene	SD	CV [% CP]	gene	SD	CV [% CP]
*ACT*	0.545	2.747	*ACT*	0.989	4.614	*ACT*	1.615	6.895
*TUA*	0.819	4.080	*TUA*	1.070	4.836	*TUA*	1.444	6.484
*UBC*	0.604	3.287	*UBC*	0.899	4.247	*UBC*	1.204	5.954
*EF1*	0.603	2.639	*EF1*	1.354	4.874	*CYP*	0.811	4.218
*CYP*	0.650	3.706	*RPL36Aa*	0.784	3.559	*PRL36Aa*	0.796	4.049
*PRL36Aa*	0.509	2.896	*UBI*	0.739	3.197	*PP2A*	1.485	6.407
*PP2A*	0.646	3.182	*PP2A*	1.028	4.153	*UBI*	0.775	3.372
*UBI*	0.526	2.327	*CACS*	0.925	3.634	*CACS*	0.992	4.419
*CACS*	0.589	2.706	*PDF2*	1.334	4.304	*F-BOX*	1.276	4.606
*UBQ*	0.557	3.123	*UBQ*	1.006	4.763	*PDF2*	1.441	4.452

SD, standard deviation; CV, coefficient of variance.

Based on BestKeeper program, in treatment 1, the stability sequence of the ten tested candidate reference genes was *PRL36Aa* > *UBI* > *ACT* > *UBQ* > *CACS* > *EF1* > *UBC* > *PP2A* > *CYP* > *TUA*, among them, *PRL36Aa* gene was the most stable reference gene with the minimum value of SD compared with other genes (**Table 3**, **Table 4**). In treatment 2, the stability order of the top ten candidate genes was: *UBI* > *PRL36Aa* > *CACS* > *UBC* > *ACT* > *UBQ* > *PP2A* > *TUA* > *PDF2* > *EF1* (**Table 3**, **Table 4**). According to the BestKeeper program analysis, the expression of *UBI*, *RPL36Aa*, *UBC*, *CACS* and *ACT* were stable with SD < 1, and *UBI* was the most stable one. In treatment 3, these genes expression stability order from high to low was: *UBI* > *PRL36Aa* > *CYP* > *CACS* > *UBC* > *F-BOX* > *PDF2* > *TUA* > *PP2A* > *ACT* (**Table 3**, **Table 4**). Among them, the expression of *UBI*, *PRL36Aa* and *CYP* were reliable and *UBI* was the most reliable one. On the contrary, the expression of *F-BOX*, *UBC*, *CACS*, *TUA*, *PP2A*, *ACT* and *PDF2* were unstable in treatment 3.

**Table 4:** Ranking order of candidate reference genes in all samples in cucumber plants.

	Method	1	2	3	4	5	6	7	8	9	10	11	12	13
Treatment 1	geNorm	*TUA*	*UBC*	*ACT*	*UBI*	*EF1*	*UBQ*	*CACS*	*PP2A*	*PRL36Aa*	*CYP*	*PDF2*	*YSL8*	*F-BOX*
	Normfinder	*EF1*	*UBQ*	*UBI*	*ACT*	*CACS*	*UBC*	*CYP*	*TUA*	*PRL36Aa*	*PP2A*	*PDF2*	*YSL8*	*F-BOX*
	BestKeeper	*PR36Aa*	*UBI*	*ACT*	*UBQ*	*CACS*	*EF1*	*UBC*	*PP2A*	*CYP*	*TUA*			
	Delta CT	*EF1*	*UBC*	*ACT*	*TUA*	*CACS*	*PP2A*	*PRL36Aa*	*UBI*	*CYP*	*UBQ*	*PDF2*	*YSL8*	*F-BOX*
	Final ranking	*EF1*	*UBI*	*ACT*	*TUA*	*CACS*	*UBC*	*PRL36Aa*	*PP2A*	*CYP*	*UBQ*	*PDF2*	*YSL8*	*F-BOX*
Treatment 2	geNorm	*UBC*	*PRL36Aa*	*UBI*	*ACT*	*TUA*	*CACS*	*PP2A*	*PDF2*	*EF1*	*UBQ*	*F-BOX*	*CYP*	*YLS8*
	Normfinder	*UBI*	*ACT*	*CACS*	*UBC*	*PRL36Aa*	*TUA*	*PP2A*	*UBQ*	*PDF2*	*EF1*	*F-BOX*	*CYP*	*YLS8*
	BestKeeper	*UBI*	*PRL36Aa*	*CACS*	*UBC*	*ACT*	*UBQ*	*PP2A*	*TUA*	*PDF2*	*EF1*			
	Delta CT	*UBI*	*ACT*	*PP2A*	*CACS*	*UBC*	*RPL36Aa*	*TUA*	*UBQ*	*EF1*	*PDF2*	*F-BOX*	*CYP*	*YLS8*
	Final ranking	*UBI*	*RPL36Aa*	*UBC*	*ACT*	*CACS*	*PP2A*	*UBQ*	*TUA*	*EF1*	*PDF2*	*F-BOX*	*CYP*	*YLS8*
Treatment 3	geNorm	*UBC*	*PP2A*	*CACS*	*TUA*	*F-BOX*	*CYP*	*UBI*	*ACT*	*PRL36aA*	*PDF2*	*UBQ*	*YSL8*	*EF1*
	Normfinder	*CACS*	*UBC*	*PP2A*	*TUA*	*CYP*	*F-BOX*	*UBI*	*ACT*	*PRL36Aa*	*PDF2*	*UBQ*	*YSL8*	*EF1*
	BestKeeper	*UBI*	*PRL36Aa*	*CYP*	*CACS*	*UBC*	*F-BOX*	*PDF2*	*TUA*	*PP2A*	*ACT*			
	Delta CT	*CACS*	*UBC*	*CYP*	*PP2A*	*TUA*	*UBI*	*F-BOX*	*ACT*	*PRL36Aa*	*PDF2*	*UBQ*	*YSL8*	*EF1*
	Final ranking	*CACS*	*CYP*	*UBI*	*UBC*	*PRL36Aa*	*PP2A*	*TUA*	*F-BOX*	*ACT*	*PDF2*	*UBQ*	*YSL8*	*EF1*

1-13: the ranking order from better to good to average.

### Comprehensive evaluation of the expression stability of candidate reference genes

Three specialized analysis programs, NormFinder, GeNorm, and BestKeeper, gave the similar but slightly different ranks for thirteen tested candidate genes. To normalize the gradate, a comprehensive analysis that regraded the expression stability of thirteen tested genes was performed. RefFinder was selected to make the comprehensive analysis. According to RefFinder analysis, in treatment 1, the order of candidate reference gene stability was: *EF1* > *UBI* > *ACT* > *TUA* > *CACS* > *UBC* > *PRL36Aa* > *PP2A* > *CYP* > *UBQ* > *PDF2* > *YSL8* > *F-BOX* (**Table 4**). Among thirteen genes, *EF1* was the most stable reference gene and *F-BOX* was the most unstable one compare with the others candidate genes. In treatment 2, the order of candidate reference gene was: *UBI* > *PRL36Aa* >*UBC* > *ACT* > *CACS* > *PP2A* > *UBQ* > *TUA* > *EF1* > *PDF2* > *F-BOX* > *CYP* > *YLS8* (**Table 4**). Among them, *UBI* was the most reliable reference gene, while *YLS8* was the most unreliable gene. In treatment 3, the reliability sequence was: *CACS* > *CYP* > *UBI* > *UBC* > *PRL36Aa* > *PP2A* > *TUA* > *F-BOX* > *ACT* > *PDF2* > *UBQ* > *YSL8* > *EF1* (**Table 4**), *CACS* was the most stable one, while *EF1* was the worst one compared with other candidate genes. These results indicated that *UBI* and *CACS* were relatively stable genes compared with other candidate genes and they could be extensive used in three different treatments.

### Validation of selected candidate reference genes

**Figure 5:**
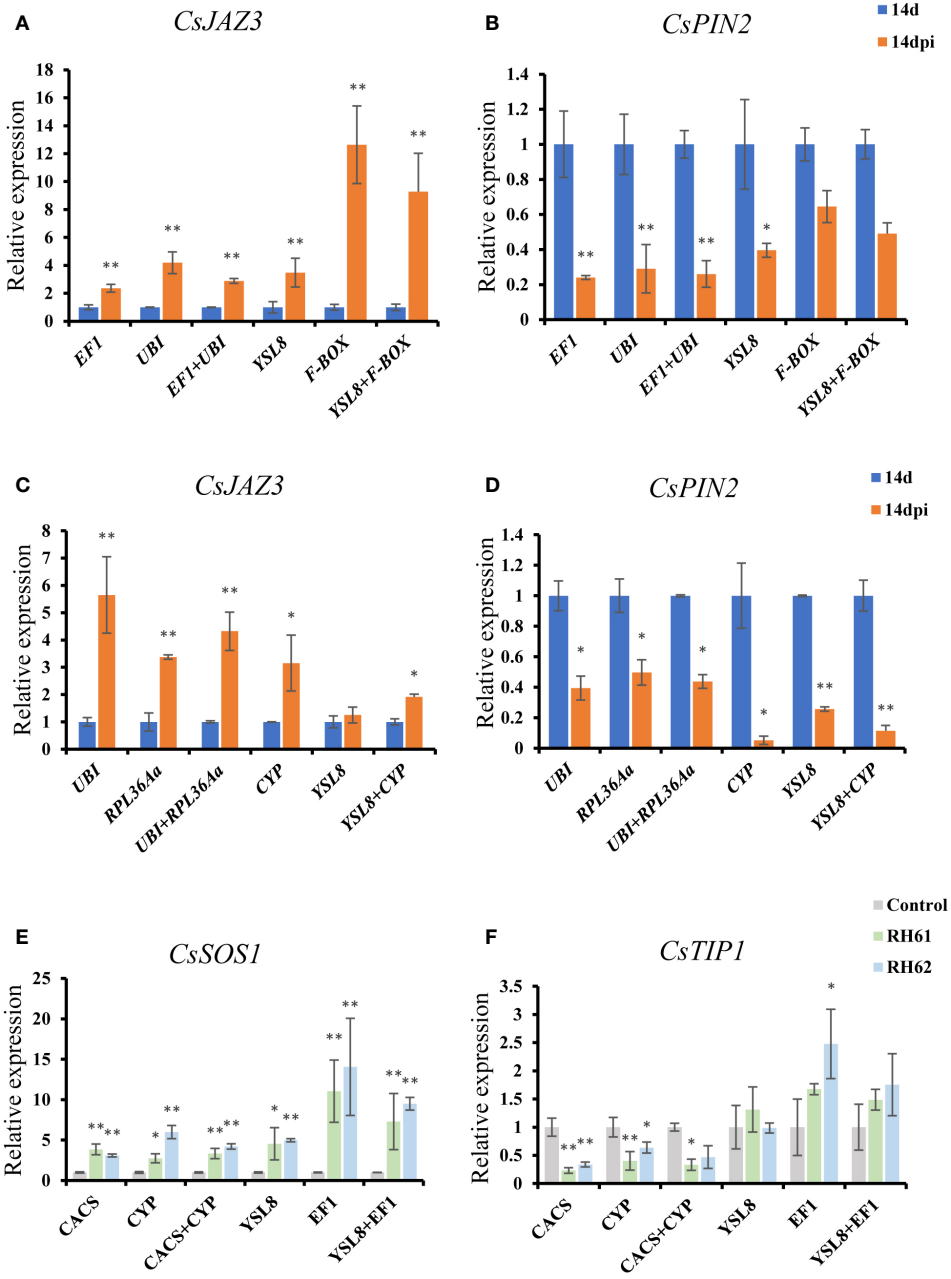
Profiling of *CsJAZ3*, *CsPIN2*, *CsTIP1* and *CsSOS1* expression in cucumber roots of treatment 1, 2 and 3 using the most suitable and least suitable sets of candidate reference genes. **(A, B)**, normalized *CsJAZ3* and *CsPIN2* expression using the most suitable candidate reference genes (*EF1* and *UBI*) and the least suitable candidate genes (*YSL8* and *F-BOX*) in treatment 1; **(C, D)**, normalized *CsJAZ3* and *CsPIN2* expression using the most suitable candidate reference genes (*UBI* and *RPL36Aα*) and the least suitable candidate genes (*YSL8* and *CYP*) in treatment 2; **(E, F)**, normalized *CsTIP1* and *CsSOS1* expression using the most suitable candidate reference genes (*CACS* and *CYP*) and the least suitable candidate genes (*YSL8* and *EF1*) in treatment 3. Abbreviation: 14 d, 14 day; 14 dpi, 14 days post infection by *M. incognita*. RH61 and RH62 are two kinds of *Pseudomonas*. "*"and"**" indicate a significant difference from the those based on t-test, with a P-value of 0.05 and 0.01 respectively.

To validate the suitability of the suitable reference gens, the top two and the bottom two ranked genes following RefFinder analysis were selected to generate an expression profile for two genes in each one treatment. *CsJAZ3* and *CsPIN2* expression were quantified across all root knots and whole roots samples infected by *M. incognita* using selected candidate reference genes (the top two: *EF1* and *UBI* in treatment 1, *UBI* and *PRL36Aa* in treatment 2; the bottom two: *YSL8* and *F-BOX* in treatment 1, *CYP* and *YLS8* in treatment 2); *CsTIP1* and *CsSOS1* expression were quantified in whole root samples inoculated by *Pseudomonas* through the top two (*CACS* and *CYP*) and the bottom two (*YSL8* and *EF1*). When applying the two best candidate reference genes for expression normalization, qRT-PCR revealed that *CsJAZ3* and *CsPIN2* transcript abundance increased and decreased, respectively, in root knot and whole root samples at 14-day after *M. incognita* infection (**Figure 5A, C**); *CsTIP1* and *CsSOS1* expressions were decreased and increased, respectively, in whole roots inoculated by *Pseudomonas*. When the two best candidate reference genes combination was used to normalize *CsJAZ3*/*CsPIN2* and *CsTIP1*/*CsSOS1* expression in cucumber roots treated by *M. incognita* and *Pseudomonas*, respectively, similar expression profiles were observed (**Figure 5A, C**).

Besides, the least stably candidate genes ranked by RefFinder also be used for qRT-PCR normalization analysis. Interestingly, when *YSL8* as reference gene to normalize the expression of *CsJAZ3* and *CsPIN2*, the results were similar with the top two candidate reference genes used in treatment 1 and 2; in treatment 1, when *F-BOX* as reference gene, *CsJAZ3* expression increased sharply and *CsPIN2* expression showed no change in root knot samples (**Figure 5B**); in treatment 2, *CsJAZ3* and *CsPIN2* expression profile showed similar result with the top two candidate reference genes when used *CYP* individual and *CYP/YSL8* combination as reference genes for normalization (**Figure 5D**); in treatment 3, when *YSL8* and *EF1* as candidate reference genes, *CsTIP1* expression had no change with or without *Pseudomonas* treatment, while *CsSOS1* transcript level was increased like the results of CACS/CYP as reference genes (**Figure 5E, F**), and similar results were also showed when using *YSL8* and *EF1* combination as reference genes for normalization (**Figure 5E, F**).

## Discussion

Cucumber as an economical vegetable is widely cultivated worldwide and is subject to serious nematode infection, especially from *M. incognita*. Plant could employ beneficial soil bacteria in the rhizosphere to enhance plant adaptability to various stresses, and *Pseudomonas* had been confirmed that play a role in increasing plant tolerance to abiotic stress (Li et al., 2021). It is important to add the knowledge regarding the response mechanism of plant to infection by *M. incognita* and the regulation mechanism by beneficial soil bacteria *Pseudomonas*. qRT-PCR is an important and convenient tool for detecting gene expression and gene function. However, selecting appropriate reference genes is necessary to ensure the stability and accuracy of qRT-PCR. In this study, the optimal reference genes in cucumber under *M. incognita* stress and *Pseudomonas* treatment were first calculated and confirmed.

Thirteen candidate reference genes *ACT* (*Actin*), *TUA* (*alpha*-*tubulin*), *UBC* (*Ubiquitin conjugating enzyme*), *EF1* (*Elongation factor 1-alpha*), *CYP* (*Cyclophilin*), PRL36Aa (*60S ribosomal protein L36a/L44*), *PP2A* (*protein phosphatase 2A regulatory subunit A*), *UBI* (*Ubiquitin-like protein*), *CACS* (*Clathrin adaptor comple subunit*), *UBQ* (*Polyubiquitin*), *F-BOX* (*F-box protein*), *YSL8* (*mitosis protein*), *PDF2* (*Protein phosphatase 2*) were selected in this study (Warzybok and Migocka, 2013; Li et al., 2013; Gan et al., 2017). The stability orders of candidate reference genes were showed according to four

The stability of candidate reference genes was analyzed according to four algorithms and a comprehensive order regraded the expression stability of thirteen tested genes was showed. In treatment 1, *EF1* and *UBI* were the top two suitable candidate reference genes, and *YSL8* and *F-BOX* were the least suitable genes according to RefFinder analysis. Thus *EF1*, *UBI*, *YSL8* and *F-BOX* were selected as reference genes to generate expression profiling for *CsJAZ3* and *CsPIN2*. The results showed that the expression trend of *CsJAZ3* and *CsPIN2* were consistent when using *EF1*, *UBI*, *YSL8* as reference genes, while their expression is different when *F-BOX* as reference gene (**Figure 5**). In addition, the result that *CsJAZ3* and *CsPIN2* expression were increased and decreased, respectively, is consistent with their orthologous gene expression in tomato and Arabidopsis after *M. incognita* infection (Hammes et al., 2005; Shukla et al., 2018). *YSL8* was the bottom two in the order of thirteen candidate genes in treatment 1, however, the M value of *YSL8* evaluated by geNorm was less than 1.5 which is a stability evaluation value. It is reasonable to speculate that, except *F-BOX*, other twelve candidate genes might be used as HKG in treatment 1 experiment. In treatment 2, *CYP* and *YSL8* were the bottom two candidate genes according to comprehensive analysis, the result was different when *CYP* as reference gene for normalization (**Figure 5**). Although the expression trend of *CsJAZ3* and *CsPIN2* was correspond when the top two candidate genes (*UBI* and RPL36A*a*) as reference gene and when *YSL8* as reference gene to normalize, *YSL8* still was not a good choice because *YSL8* even ranked latter than *CYP* and its M value is bigger than 1.5 which represents YSL8 is an unreliable reference gene. In addition, *ACT* are suitable reference genes in root knot and whole root samples after *M. incognita* infection, but they were not suitable for a reference gene under *Pseudomonas* treatment (**Table 4**, **Figure 3**). In previous research, *ACT* has been employed to normalize gene expression in several species infected by nematode, including Arabidopsis and tomato (Hewezi et al., 2010; Uehara et al., 2010; Vijayapalani et al., 2018; Xu et al., 2019; Huang et al., 2022), although none of the research involves HKGs screening in nematode infection experiment. It might be reasonable to speculate that *ACT* can be used as a universal HKG to normalize gene expression level in different species under nematode infection treatment.

*Pseudomonas* had been certified that could enhance plant adaptability to salt stress through inducing the production of stress alleviating metabolites, thus two salt-response related genes, *CsTIP1* (aquaporin protein) and *CsSOS1* (salt overly sensitive gene), were selected to identify the stability of candidate reference genes. In treatment 3, M values of thirteen candidate genes were less than 1.5, however, the bottom two genes (*YSL8* and *EF1*) as reference genes for normalization showed different expression of *CsTIP1*and *CsSOS1* compared with the top two candidate genes (*CACS* and *CYP*). Different from above result, *EF1* could as a suitable gene in *Arabidopsis thaliana*, *Nicotiana benthamiana* and *Solanum tuberosum* under abiotic stress including heat, cold, hormones, dehydration and biotic stress including *Pseudoperonospora cubensis* (Nicot et al., 2005; Remans et al., 2008; Catinot et al., 2008). *EF1* in cucumber is also the most reliable expression of reference gene in leaves, stems and roots under *Cucumber green mottle mosaic virus* (CGMMV) treatment (Liang et al., 2018). When *CACS* and *CYP* as reference genes to normalize the expression of *CsTIP1* and *CsSOS1*, the results were consistent with that *TIP1* and *SOS1* were reduced and induced, respectively, by salt stress (Zhu et al., 2009; Zhang et al., 2020), and the result also provided molecular evidence for *Pseudomonas* inoculation could improve plant tolerance for salt stress. These results suggested that HKG cannot be utilized universally across different plant species, experimental conditions, or even different environmental conditions with a single species.

Recently, new reference gene *CsARF* (ADP ribosylation factor 1) in cucumber had been identified, its expression was stable and could be a suitable housekeeping gene in cucumber stems infected by *Pectobacterium Brasiliense* (Yuan et al., 2022). In addition to those identified reference genes, continuing exploration and validation of new housekeeping genes being pushed forward for different organs, various developmental stages, under different environmental stresses of plants, thus data acquired by qRT-PCR would be more accuracy and reliable.

In conclusion, *EF1* is the most suitable reference gene in the root knot samples of cucumber infected by root knot nematode, although *EF1* has never been considered in previous plant-nematode studies. *UBI* is the most suitable reference gene in the whole root samples of cucumber under root-knot nematode stress, and *CACS* is the most suitable reference gene in the whole root samples of cucumber treated by *Pseudomonas*. Through BestKeeper, NormFinder, and geNorm analysis, *UBI*, *ACT* and *CACS* were used simultaneously as reference genes no matter in whole root or root knot samples of cucumber infected by *M. incognita*. *CACS*, *CYP* and *UBI* were suitable reference genes in *Pseudomonas* treated cucumber whole root samples. Among them, *UBI* and *CACS* are relatively stable and could be utilized in three experiments conditions.

## Data availability statement

The raw data supporting the conclusions of this article will be made available by the authors, without undue reservation.

## Author contributions

YT and LG conceived and designed the experiments. TJ, SM, XW and ML performed the experiments and collected the data. TJ and SM executed the data analyses. All authors contributed to the interpretation of the results. TJ, SM, YT and LG wrote the manuscript. All authors contributed to the article and approved the submitted version.

## References

[B1] Andersen C. L. Jensen J. L. Orntoft T. F. (2004). Normalization of real-time quantitative reverse transcription-PCR data: a model-based variance estimation approach to identify genes suited for normalization, applied to bladder and colon cancer data sets. Cancer Res. 64, 5245–5250. doi: 10.1158/0008-5472.CAN-04-0496 15289330

[B2] Catinot J. Buchala A. Abou-mansour E. Métraux J. P. (2008). Salicylic acid production in response to biotic and abiotic stress depends on isochorismate in *Nicotiana benthamiana* . FEBS Lett. 582, 473–478. doi: 10.1016/j.febslet.2007.12.039 18201575

[B3] Chen W. Shao J. Ye M. Yu K. Bednarek S. Duan X.. (2017). Blueberry VcLON 2, a peroxisomal LON protease, is involved in abiotic stress tolerance. Environ. Exp. Bot. 134, 1–11. doi: 10.1016/j.envexpbot.2016.10.008

[B4] Duan M. Wang J. Zhang X. Yang H. Wang H. Qiu Y.. (2017). Identification of optimal reference genes for expression analysis in radish (*Raphanus sativus* l.) and its relatives based on expression stability. Front. Plant Sci. 8, 1605. doi: 10.3389/fpls.2017.01605 28966627 PMC5605625

[B5] Gan D. Zhan M. Yang F. Zhang Q. Hu K. Xu W.. (2017). Expression analysis of argonaute, dicer-like, and RNA-dependent RNA polymerase genes in cucumber (*Cucumis sativus* l.) in response to abiotic stress. J. Genet. 96 (2), 235–249. doi: 10.1007/s12041-017-0758-y 28674223

[B6] Hammes U. Z. Schachtman D. P. Berg R. H. Nielsen E. Koch W. McIntyre L. M.. (2005). Nematode-induced changes of transporter gene expression in arabidopsis roots. Mol. Plant-Microbe Interact. 18 (12), 1247–1257. doi: 10.1094/MPMI-18-1247 16478044

[B7] Hewezi T. Howe P. J. Maier T. R. Hussey R. S. Mitchum M. G. Davis E. L.. (2010). Arabidopsis spermidine synthase is targeted by an effector protein of the cyst nematode *Heterodera schachtii* . Plant Physiol. 152 (2), 968–984. doi: 10.1104/pp.109.150557 19965964 PMC2815906

[B8] Huang S. Li R. Zhang Z. Li L. Gu X. Fan W.. (2009). The genome of the cucumber, *Cucumis sativus* l. Nat. Genet. 41, 1275–1281. doi: 10.1038/ng.475 19881527

[B9] Huang H. Zhao W. Qiao H. Li C. Sun L. Yang R.. (2022). *SlWRKY45* interacts with jasmonate-ZIM domain proteins to negatively regulate defense against the root-knot nematode *Meloidogyne incognita* in tomato. Horticult. Res. 9, uhac197. doi: 10.1093/hr/uhac197 PMC9630973 36338841

[B10] Jaiteh F. Kwoseh C. Akromah R. (2012). Evaluation of tomato genotypes for resistance to root-knot nematodes. Afr. Crop Sci. J. 20, 41–49. doi: 10.4314/ACSJ.V20I1

[B11] Jones L. M. De Giorgi C. Urwin P. E. (2011). “C. elegans as a resource for studies on plant-parasitic nematodes,” in Genomics and molecular genetics of plant–nematode interactions. Eds. Gheysen G. Fenoll C. Jones J. T. (UK: Springer Science and Business Media, B.V), 175–220. doi: 10.1007/978-94-007-0434-3_10

[B12] Joseph J. T. Poolakkalody N. J. Shah J. M. (2018). Plant reference genes for development and stress response studies. J. Biosci. 43 (1), 173–187. doi: 10.1007/s12038-017-9728-z 29485125

[B13] Kopczewski T. Kuźniak E. Kornaś A. Rut G. Nosek M. Ciereszko I.. (2022). Local and systemic changes in photosynthetic parameters and antioxidant activity in cucumber challenged with *Pseudomonas syringae* pv lachrymans. Int. J. Mol. Sci. 21 (17), 6378. doi: 10.3390/ijms21176378 PMC7504232 32887449

[B14] Kumar M. Etesami H. Kumar V. (2019). Saline soil-based agriculture by halotolerant microorganisms (Singapore: Springer Nature Singapore Pte Ltd).

[B15] Le D. T. Aldrich D. L. Valliyodan B. Watanabe Y. Ha C.V. Nishiyama. R. (2012). Evaluation of candidate reference genes for normalization of quantitative RT-PCR in soybean tissues under various abiotic stress conditions. PloS One 7, e46487. doi: 10.1371/annotation/6a5108f5-50f8-418e-854d-8f3eb94e6fc0 23029532 PMC3460875

[B16] Liang C. Hao J. Meng Y. Luo L. Li J. (2018). Identifying optimal reference genes for the normalization of microRNA expression in cucumber under viral stress. PloS One 13 (3), e0194436. doi: 10.1371/journal.pone.0194436 29543906 PMC5854380

[B17] Li P. Chen L. Zhou Y. Xia X. Shi K. Chen Z.. (2013). Brassinosteroids-induced systemic stress tolerance was associated with increased transcripts of several defence-related genes in the phloem in *Cucumis sativus* . PloS One 8, 1–8. doi: 10.1371/journal.pone.0066582 PMC3686678 23840504

[B18] Li H. La S. Zhang X. Gao L Tian Y. (2021). Salt-induced recruitment of specific root-associated bacterial consortium capable of enhancing plant adaptability to salt stress. Multidiscip. J. Microbial. Ecol. J. 15 (10), 2865–2882. doi: 10.1038/s41396-021-00974-2 PMC8443564 33875820

[B19] Li X. Ma S. Shan N. Zhang X. L. Sui X. L. Zhang Z. X.. (2017). A protocol for agrobacterium-mediated transformation of cucumber (*Cucumis sativus* l.) from cotyledon explants. Protocol. exch. 6159, 1–19. doi: 10.1038/protex.2017.107

[B20] Lü J. Sui X. Ma S. Li X. Liu H. Zhang Z.. (2017). Suppression of cucumber stachyose synthase gene (CsSTS) inhibits phloem loading and reduces low temperature stress tolerance. Plant Mol. Biol. 95 (1-2), 1–15. doi: 10.1007/s11103-017-0621-9 28608281 PMC5594042

[B21] Ma S. Sun L. L. Sui X. L. Li Y. Chang Y. Fan J.. (2019). Phloem loading in cucumber: combined symplastic andapoplastic strategies. Plant J. 98, 391–404. doi: 10.1111/tpj.14224 30604489

[B22] Mbaluto C. M. Ahmad E. M. Fu M. Martínez-Medina A. van Dam N. M. (2020). The impact of spodoptera exigua herbivory on *Meloidogyne incognita* induced root responses depends on the nematodeslife cycle stages. AoB Plants 12, plaa029. doi: 10.1093/aobpla/plaa029 32665829 PMC7336558

[B23] Miao L. Qin X. Gao L. H. Li Q. Li S. He C.. (2019). Selection of reference genes for quantitative real-time PCR analysis in cucumber (*Cucumis sativus* l.), pumpkin (*Cucurbita moschata* duch.) and cucumber-pumpkin grafted plants. Peer J. 7, e6536. doi: 10.7717/peerj.6536 31024757 PMC6475253

[B24] Migocka M. Papierniak A. (2011). Identification of suitable reference genes for studying gene expression in cucumber plants subjected to abiotic stress and growth regulators. Mol. Breed. 28 (3), 343–357. doi: 10.1007/s11032-010-9487-0

[B25] Moens M. Perry R. N. (2009). Migratory plant endoparasitic nematodes: a group rich in contrasts and divergence. Annu. Rev. Phytopathol. 47, 313–332. doi: 10.1146/annurev-phyto-080508-081846 19400647

[B26] Nicot N. Hausman J. F. Hoffmann L. Evers D. (2005). Housekeeping gene selection for real-time RT-PCR normalization in potato during biotic and abiotic stress. J. Exp. Bot. 56, 2907–2291. doi: 10.1093/jxb/eri285 16188960

[B27] Niu H. Liu X. Tong C. Wang H. Li S. Lu L.. (2018). The WUSCHEL-related homeobox1 gene of cucumber regulates reproductive organ development. J. Exp. Bot. 69 (22), 5373–5387. doi: 10.1093/jxb/ery329 30204887

[B28] Nolan T. Hands R. E. Bustin S. A. (2006). Quantification of mRNA using real-time RT- PCR. Nat. Protoc. 1, 1559–1582. doi: 10.1038/nprot.2006.236 17406449

[B29] Olaetxea M. Mora V. Bacaicoa E. Garnica M. Fuentes M. Casanova E.. (2015). Abscisic acid regulation of root hhydraulic conductivity and aquaporin gene expression is crucial to the plant shoot growth eenhancement caused by rhizosphere humic acids. Plant Physiol. 169 (4), 2587–2596. doi: 10.1104/pp.15.00596 26450705 PMC4677878

[B30] Petriccione M. Mastrobuoni F. Zampella L. Scortichini M. (2015). Reference gene selection for normalization of RT-qPCR gene expression data from actinidia deliciosa leaves infected with *Pseudomonas syringae* pv. Sci. Rep. 5, 1696. doi: 10.1038/srep16961 PMC4652207 26581656

[B31] Pfaffl M. W. Tichopad A. Prgomet C. Neuvians T. P. (2004). Determination of stable housekeeping genes, differentially regulated target genes and sample integrity: best-keeper- excel-based tool using pair-wise correlations. Biotechnol. Lett. 26, 509–515. doi: 10.1023/B:BILE.0000019559.84305.47 15127793

[B32] Qin N. Gao Y. Cheng X. Yang Y. Wu J. Wang J.. (2021). Genome-wide identification of CLE gene family and their potential roles in bolting and fruit bearing in cucumber (Cucumis sativus l.). BMC Plant Biol. 21 (1), 143. doi: 10.1186/s12870-021-02900-2 33740893 PMC7980335

[B33] Rajsz A. Warzybok A. Migocka M. (2016). Genes encoding cucumber full-size ABCG proteins show different responses to plant growth regulators and sclareolide. Plant Mol. Biol. Rep. 34, 720–736. doi: 10.1007/s11105-015-0956-9 PMC4923091 27429510

[B34] Remans T. Smeets K. Opdenakker K. Mathijsen D. Vangronsveld J. Cuypers A. (2008). Normalisation of real-time RT-PCR gene expression measurements in *Arabidopsis thaliana* exposed to increased metal concentrations. Planta 227, 1343–1349. doi: 10.1007/s00425-008-0706-4 18273637

[B35] Rodrigues F. Marcelino-guimaraes F. C. Lima A. Abdelnoor R. V. Margis R. (2010). The use of microRNAs as reference genes for quantitative polymerase chain reaction in soybean. Anal. Biochem. 406, 185–192. doi: 10.1016/j.ab.2010.07.020 20670612

[B36] Sandhya V. Ali Sk Z. Grover M. Reddy G. Venkateswarlu B. (2010). Effect of plant growth promoting pseudomonas spp. 0n compatible solutes antioxidant status and plant growth of maize under drought stress. Plant Growth Regul. 62, 21–30. doi: 10.1007/s10725-010-9479-4

[B37] Shahid M. Gowen S. R. Burhan M. (2022). Studies on the possible role of plant host on the development of root-knot nematode, *Meloidogyne javanica* and *Pasteuria penetrans* as affected by different harvesting dates. Plant Prot. 6 (2), 133–141. doi: 10.33804/pp.006.02.4207

[B38] Shen J. Zhang Y. Ge D. Wang Z. Song W. Gu R.. (2019). CsBRC1 inhibits axillary bud outgrowth by directly repressing the auxin efflux carrier CsPIN3 in cucumber. Proc. Natl. Acad. Sci. U S A. 116 (34), 17105–17114. doi: 10.1073/pnas.1907968116 31391306 PMC6708385

[B39] Shukla N. Yadav R. Kaur P. Rasmussen S. Goel S. Agarwal M.. (2018). Transcriptome analysis of root-knot nematode (*Meloidogyne incognita*)-infected tomato (*Solanum lycopersicum*) roots reveals complex gene expression profiles and metabolic networks of both host and nematode during susceptible and resistance responses. Mol. Plant Pathol. 19 (3), 615–633. doi: 10.1111/mpp.12547 28220591 PMC6638136

[B40] Song Y. Wang Y. Guo D. Jing L. (2019). Selection of reference genes for quantitative real-time PCR normalization in the plant pathogen *Puccinia rasiliens schw* . BMC Plant Biol. 19 (1), 20. doi: 10.1186/s12870-019-1629-x 30634896 PMC6329156

[B41] Sun L. Sui X. Lucas W. J. Li Y. Feng S. Ma S.. (2019). Down-regulation of the sucrose transporter CsSUT1 causes male sterility by altering carbohydrate supply. Plant Physiol. 180 (2), 986–997. doi: 10.1104/pp.19.00317 30967482 PMC6548282

[B42] Uehara T. Sugiyama S. Matsuura H. Arie T. Masuta C. (2010). Resistant and susceptible responses in tomato to cyst nematode are differentially regulated by salicylic acid. Plant Cell Physiol. 51 (9), 1524–1536. doi: 10.1093/pcp/pcq109 20660227

[B43] Vandesompele J. De Preter K. Pattyn F. Poppe B. Van Roy N. De Paepe A.. (2002). Accurate normalization of real-time quantitative RT-PCR data by geometric averaging of multiple internal control genes. Genome Biol. 3 (0034), 1–11. doi: 10.1186/gb-2002-3-7-research0034 PMC126239 12184808

[B44] Vejan P. Abdullah R. Khadiran T. Ismail S. Nasrulhaq Boyce A. (2016). Role of plant growth promoting rhizobacteria in agricultural sustainability-a review. Molecules 21 (5), 573. doi: 10.3390/molecules21050573 27136521 PMC6273255

[B45] Vijayapalani P. Hewezi T. Pontvianne F. Baum T. J. (2018). An effector from the *Cyst nematode heterodera schachtii* derepresses host rRNA genes by altering histone acetylation. Plant Cell. 30 (11), 2795–2812. doi: 10.1105/tpc.18.00570 30333146 PMC6305986

[B46] Wang J. Pan C. Wang Y. Ye L. Wu J. Chen L.. (2015). Genome-wide identification of MAPK, MAPKK, and MAPKKK gene families and transcriptional profiling analysis during development and stress response in cucumber. BMC Genomics 16 (1), 386. doi: 10.1186/s12864-015-1621-2 25976104 PMC4432876

[B47] Warzybok A. Migocka M. (2013). Reliable reference genes for normalization of gene expression in cucumber grown under different nitrogen nutrition. PloS One 8 (9), e72887. doi: 10.1371/journal.pone.0072887 24058446 PMC3772881

[B48] Wei G. Tian P. Zhang F. Qin H. Miao H. Chen Q.. (2016). Integrative analyses of nontargeted volatile profiling and transcriptome data provide molecular insight into VOC diversity in cucumber plants (*Cucumis sativus*). Plant Physiol. 172 (1), 603–618. doi: 10.1104/pp.16.01051 27457123 PMC5074635

[B49] Wen C. Zhao W. Liu W. Yang L. Wang Y. Liu X.. (2019). CsTFL1 inhibits determinate growth and terminal flower formation through interaction with CsNOT2a in cucumber. Development 146 (14), dev180166. doi: 10.1242/dev.180166 31320327 PMC6679365

[B50] Xu X. Fang P. Zhang H. Chi C. Song L. Xia X.. (2019). Strigolactones positively regulate defense against root-knot nematodes in tomato. J. Exp. Bot. 70 (4), 1325–1337. doi: 10.1093/jxb/ery439 30576511 PMC6382333

[B51] Yook K. Harris T. W. Tamberlyn B. Cabunoc A. Chan J. Chen W. J.. (2011). Wormbase 2012: more genomics, more data, new website. Nucleic Acids Res. 40, 735–741. doi: 10.1093/nar/gkr954 PMC3245152 22067452

[B52] Yuan L. Zhao Y. Xie H.. (2022). Selection and evaluation of suitable reference genes for quantitative gene expression analysis during infection of *Cucumis sativus* with *Pectobacterium rasiliense* . J. Appl. Microbiol. 132 (5), 3717–3734. doi: 10.1111/jam.15481 35138009

[B53] Zhang H. Li S. Yang L. Cai G. Chen H. Gao D.. (2021). Gain-of-function of the 1-aminocyclopropane-1-carboxylate synthase gene ACS1G induces female flower development in cucumber gynoecy. Plant Cell. 33 (2), 306–321. doi: 10.1093/plcell/koaa018 33793793 PMC8136878

[B54] Zhang H. Murzello C. Sun Y. Kim M. S. Xie X. Jeter R. M.. (2010). Choline and osmotic-stress tolerance induced in arabidopsis by the soil microbe *Bacillus subtilis* (GB03). Mol. Plant Microbe Interact. 23 (8), 1097–1104. doi: 10.1094/MPMI-23-8-1097 20615119

[B55] Zhang F. Qin Z. Zhou X. Xin M. Li S. Luan J.. (2019). Expression and functional analysis of the propamocarb-related gene CsMAPEG in cucumber. BMC Plant Biol. 19 (1), 371. doi: 10.1186/s12870-019-1971-z 31438856 PMC6704574

[B56] Zhang T. Shi Z. Zhang X. Zheng S. Wang J. Mo J.. (2020). Alleviating effects of exogenous melatonin on salt stress in cucumber. Sci. Hortic. 262, 109070. doi: 10.1016/j.scienta.2019.109070

[B57] Zhao J. Jiang L. Che G. Pan Y. Li Y. Hou Y.. (2019) A Functional Allele of CsFUL1 Regulates Fruit Length through Repressing CsSUP and Inhibiting Auxin Transport in Cucumber. Plant Cell. 31 (6), 1289–1307. doi: 10.1105/tpc.18.00905 30979795 PMC6588310

[B58] Zhou Y. Zhao D. Shuang L. Xiao D. Xuan Y. Duan Y.. (2020). Transcriptome analysis of rice roots in response to root-knot nematode infection. Int. J. Mol. Sci. 21 (3), 848. doi: 10.3390/ijms21030848 32013011 PMC7037758

[B59] Zhu Y. X. Yang L. Liu N. Yang J. Zhou X. K. Xia Y. C.. (2009). Genome-wide identification, structure characterization, and expression pattern profiling of aquaporin gene family in cucumber. BMC Plant Biol. 19 (1), 1–23. doi: 10.1186/s12870-019-1953-1 PMC6686268 31390991

