# Supplementary material for: Reference genes identification for qRT-PCR normalization of gene expression analysis in Cucumis sativus under Meloidogyne incognita infection and Pseudomonas treatment
Source: Front Plant Sci. 2022 Dec 15;13:1061921. doi: 10.3389/fpls.2022.1061921 (PMC9799720; doi:10.3389/fpls.2022.1061921)
Supplement: Supplementary file 1 [file Table_1.docx]

Supplement table 1 The Ct values of 13 candidate reference genes

| Genes | Treatment 1 | | |  | Treatment 2 | | |  | Treatment 3 | | |
| --- | --- | --- | --- | --- | --- | --- | --- | --- | --- | --- | --- |
|  | Average Ct | SD | Difference |  | Average Ct | SD | Difference |  | Average Ct | SD | Difference |
| *ACT* | 19.85 | 0.83 | 4.64 |  | 21.42 | 1.20 | 4.81 |  | 23.43 | 1.77 | 5.04 |
| *TUA* | 20.06 | 1.03 | 4.78 |  | 22.12 | 1.37 | 5.15 |  | 22.55 | 1.92 | 5.95 |
| *UBC* | 18.38 | 0.81 | 3.75 |  | 21.16 | 1.13 | 4.50 |  | 20.38 | 1.48 | 4.20 |
| *EF1* | 22.86 | 0.83 | 3.83 |  | 27.62 | 1.50 | 5.49 |  | 26.25 | 2.46 | 6.58 |
| *CYP* | 17.54 | 0.80 | 2.91 |  | 20.38 | 1.82 | 6.12 |  | 19.51 | 1.45 | 5.48 |
| *PRL36Aa* | 17.56 | 0.65 | 2.87 |  | 22.04 | 1.00 | 3.73 |  | 19.77 | 1.03 | 3.23 |
| *PP2A* | 20.29 | 0.81 | 3.06 |  | 24.75 | 1.30 | 5.02 |  | 23.35 | 1.73 | 4.73 |
| *UBI* | 22.58 | 0.69 | 3.14 |  | 23.11 | 0.96 | 3.98 |  | 22.98 | 0.93 | 3.73 |
| *CACS* | 21.78 | 0.72 | 2.94 |  | 25.45 | 1.18 | 4.69 |  | 24.14 | 1.41 | 4.47 |
| *UBQ* | 17.83 | 0.69 | 3.21 |  | 21.12 | 1.19 | 4.61 |  | 17.76 | 1.48 | 5.25 |
| *F-BOX* | 24.40 | 1.36 | 6.30 |  | 27.46 | 1.44 | 5.88 |  | 27.71 | 1.41 | 4.12 |
| *YSL8* | 25.84 | 1.57 | 6.60 |  | 26.53 | 1.32 | 5.23 |  | 25.03 | 1.57 | 5.40 |
| *PDF2* | 28.11 | 1.14 | 5.82 |  | 30.98 | 1.65 | 6.48 |  | 32.37 | 1.80 | 6.15 |

Difference: Maximum Ct value - minimum Ct value; SD: standard deviation.

Supplement table 2 Primers used in this study

| Gene | Gene ID in cucumber | Forward primer | Reverser primer |
| --- | --- | --- | --- |
| *CsJAZ3* | Csa7G448810 | CAGAGCCTACAACCGCTCAA | CTTCACCGGAGCTGGAGTTT |
| *CsPIN2* | Csa1G427480 | TCGTGTCGATTCCGATGTGG | AAGCTCTAGGTGTCGGCTCT |
| *CsSOS1* | Csa5G098980 | TCCAACTGATGCTGTGCTGT | GGAACTCTAGTGCCTCGCAA |
| *CsTIP1* | Csa6G448110 | GGCAACCTGTTGTAGCCAGT | GGATGAGTAGCCTCTTCGGG |
